# Is Preoperative Biochemical Testing for Pheochromocytoma Necessary for All Adrenal Incidentalomas?

**DOI:** 10.1097/MD.0000000000001948

**Published:** 2015-11-13

**Authors:** Joo Hyun Jun, Hyun Joo Ahn, Sangmin M. Lee, Jie Ae Kim, Byung Kwan Park, Jee Soo Kim, Jung Han Kim

**Affiliations:** From the Department of Anesthesiology and Pain Medicine, Samsung Medical Center, Sungkyunkwan University School of Medicine (JHJ, HJA, SML, JAK); Department of Anesthesiology and Pain Medicine, Kangnam Sacred Heart Hospital, Hallym University College of Medicine (JHJ); Department of Radiology (BKP); and Department of Surgery, Samsung Medical Center, Sungkyunkwan University School of Medicine, Seoul, Korea (JSK, JHK).

## Abstract

This study examined whether imaging phenotypes obtained from computed tomography (CT) can replace biochemical tests to exclude pheochromocytoma among adrenal incidentalomas (AIs) in the preoperative setting.

We retrospectively reviewed the medical records of all patients (n = 251) who were admitted for operations and underwent adrenal-protocol CT for an incidentally discovered adrenal mass from January 2011 to December 2012. Various imaging phenotypes were assessed for their screening power for pheochromocytoma. Final diagnosis was confirmed by biopsy, biochemical tests, and follow-up CT.

Pheochromocytomas showed similar imaging phenotypes as malignancies, but were significantly different from adenomas. Unenhanced attenuation values ≤10 Hounsfield units (HU) showed the highest specificity (97%) for excluding pheochromocytoma as a single phenotype. A combination of size ≤3 cm, unenhanced attenuation values ≤ 10 HU, and absence of suspicious morphology showed 100% specificity for excluding pheochromocytoma.

Routine noncontrast CT can be used as a screening tool for pheochromocytoma by combining 3 imaging phenotypes: size ≤3 cm, unenhanced attenuation values ≤10 HU, and absence of suspicious morphology, and may substitute for biochemical testing in the preoperative setting.

## INTRODUCTION

Adrenal incidentalomas (AIs) are adrenal masses discovered incidentally in imaging studies performed for reasons unrelated to adrenal pathology.^1^ AI is very common and estimated to occur in approximately 3% to 5% of the adult population.^1,2^ Although the rate of AI is only 0.2% for young subjects, it is as high as 7% to 10% in those older than 70 years.^3^ Thus, AIs represent a diagnostic challenge that will only become worse as the population ages and the use of computed tomography (CT) and magnetic resonance imaging (MRI) increases.

The differential diagnosis of AI includes benign adrenal adenoma (functioning and nonfunctioning), pheochromocytoma, primary adrenal carcinoma, metastasis, and other adrenal masses (myelolipoma, cyst, ganglioneuroma).^4^ Of these, unrecognized pheochromocytoma may provoke a potentially life-threatening incident during surgery. Therefore, excluding pheochromocytoma is critical in the preoperative setting.

Because up to 40% of patients with pheochromocytoma have no symptoms, recent guidelines recommend the measurement of fractionated plasma or 24-h urinary metanephrine in all cases with AI to exclude occult and asymptomatic pheochromocytoma.^4,5^ However, many surgeons do not routinely order biochemical assays preoperatively.^6^ These tests are usually provided only once a week at hospitals and take a week to get the results. Delaying operation causes many troubles and burdens to patients and hospital, and is a dilemma because most AIs are nonfunctioning adenomas.^7^ Furthermore, biochemical work-ups have their own shortcomings. Approximately, 50% of patients with small pheochromocytoma exhibit modestly abnormal or even normal test results.^8^ False-positive cases are also common. Exercise, posture, food, stress, and medications can result in modestly elevated metanephrine, bringing confusion to diagnosis.^9^

Patients already have their abdominal or chest CT before operation by which their AI was detected. Nonadenomas have distinguishable image characteristics on CT, including large size, irregular shape, areas of necrosis, calcification, and inhomogeneity.^10^ It would be very helpful if these phenotypes of CT can exclude pheochromocytoma with high specificity.

Therefore, we obtained a triphasic adrenal-protocol CT for every AI patient admitted for surgery and compared various CT phenotypes, which are known to be sensitive to detect pheochormocytoma.

The purpose of this study was to examine the diagnostic power of individual imaging phenotype obtained from CT and to determine which phenotype has the highest specificity to exclude pheochromocytoma and can replace biochemical testing in the preoperative setting.

## MATERIALS AND METHODS

### Study Population

This retrospective study was approved by our institutional review board, and the need for informed consent was waived. We investigated all AI patients who were admitted for surgery from January 2011 to December 2012.

**FIGURE 1:**
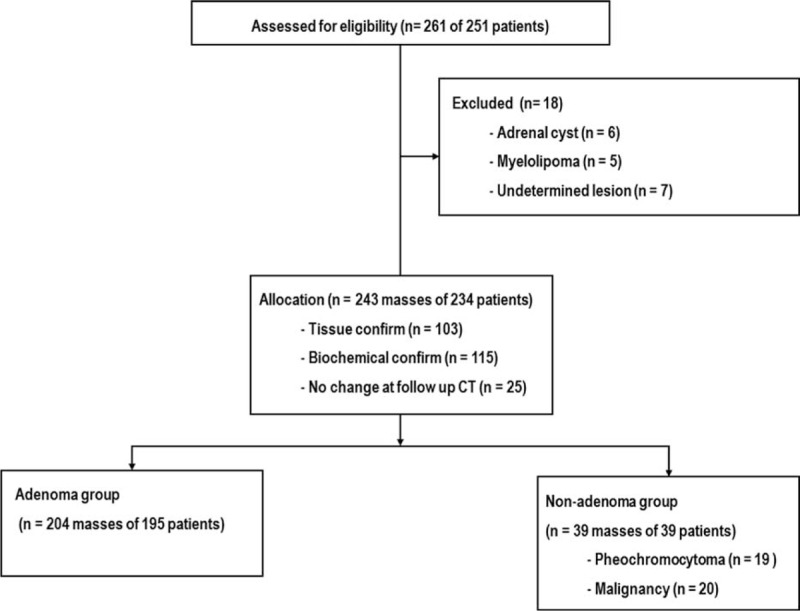
. Flow diagram of patient selection.

A total of 251 patients with 261 adrenal masses received adrenal protocol CT. If an adrenal mass had the diagnostic features of a benign lesion such as a myelolipoma or cyst, no additional work-up or follow-up imaging was needed and the patient was excluded from the study (n = 11). As a result, 250 adrenal masses were considered for entry into our study. During follow-up, another 7 adrenal masses were excluded as undetermined lesions (no six month follow-up CT or cytologic/histologic confirmation). Consequently, 243 adrenal masses in 234 patients were included in the final analysis. These consisted of 204 adenomas and 39 nonadenomas (19 pheochomocytomas and 20 malignancies). Malignancies included adrenocortical carcinoma (n = 11) and metastases (n = 9) (Fig. 1).

The final diagnosis of adenoma was confirmed by pathologic examination (n = 65), size stability at 6-month follow-up CT (n = 25), or negative plasma or urinary metanephrines (n = 114). All nonadenomas were confirmed by pathologic examination except for 1 pheochromocytoma that was confirmed by repeated biochemical testing.

### Clinical Data

Clinical data were obtained from electronic medical records. Demographic information, pre-existing medical conditions, and the presence of classic pheochromocytoma symptoms (headache, sweating, palpitations, febrile sense, or episodic hypertension) were recorded.

### Image Analysis

A triphasic adrenal-protocol CT (unenhanced, 1-minute contrast enhanced [early], and 15-minute contrast enhanced [delayed]) was performed for all patients. A total of 120 mL of contrast media was injected at a rate of 2.5 to 3 mL/sec and a triphasic image was obtained with multidetector CT (MDCT). One reviewer evaluated CT examinations and recorded the diameter of the mass, the presence of suspicious morphology (calcifications, necrotic or cystic changes, and inhomogeneity), the attenuation of the mass on unenhanced (UA), early enhanced (EA), and delayed enhanced CT (DA). The diameter of each adrenal mass was measured on the slice with the largest surface area. A region of interest (ROI) was selected in the adrenal lesion showing the strongest enhancement on early enhanced CT images. The same ROI was then located in the unenhanced and delayed phase CT images. The ROI did not cover the peripheral area of the lesion in order to avoid a partial volume effect and did not contain any calcifications, blood vessels, or necrotic or cystic areas within the lesion. The attenuation value calculated by the HU of the ROI was measured 3 times and averaged for each of the unenhanced, early, and delayed enhanced images. Necrosis was defined as a region with an EA <20 HU. Calcification was defined as a region with a UA >120 HU. The absolute washout rate (APW) and relative washout rate (RPW) were calculated as follows.^11^ 
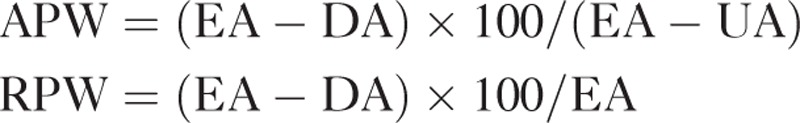


### Biochemical Testing

Laboratory results including 24-h urine or plasma fractionated metanephrines and 24-h urine vanillylmandelic acid (VMA) were evaluated. Before sampling, patients were advised to discontinue all medications that might alter urinary and plasma concentration of catecholamine or metanephrine. Urine or plasma metanephrine levels greater than 2 times the upper limit of normal were considered diagnostic of pheochromocytoma. Urine or plasma metanephrine levels between 1 and 2 times the upper limit of normal were considered equivocal or “borderline elevated” results. Urine or plasma metanephrine levels lower than the upper limit of normal ruled out the presence of a pheochromocytoma.^12^ All biochemical tests were done by high-performance liquid chromatography (HPLC) method.

### Intraoperative Hemodynamics

Hemodynamic instability was assessed as the maximum intraoperative blood pressure (BP), duration in minutes that the SBP was higher than 200 mm Hg, lowest intraoperative BP, maximum and minimum heart rates, and presence of tachycardic (≥110 beats/min) and bradycardic (≤50 beats/min) episodes. These hemodynamic parameters were selected on the basis of a previous report of patients with pheochromocytomas^13^ and served as descriptors of hemodynamic instability. Intraoperative use of vasoactive drugs was also abstracted.

### Statistical Analysis

The following statistical analyses were performed with MedCalc 7.3 for Windows (MedCalc software, Mariakerke, Belgium) or SPSS 21.0 for Windows (SPSS Inc., Chicago, IL). First, the clinical, demographic, and radiologic parameters between adenomas and nonadenomas (pheochromocytoma, malignancy) were compared using the Chi-square, Kruskal–Wallis, and Mann–Whitney tests. Second, the sensitivity, specificity, positive predictive value (PPV), and negative predictive value (NPV) for any specific cutoff value or a combination of values were calculated. Sensitivity was defined as the probability that an adrenal mass is classified as adenoma given that it is truly an adenoma. Specificity was the probability that an adrenal mass is classified as nonadenoma given that it is truly a nonadenoma. PPV was the probability that an adrenal mass with a positive test is truly an adenoma. NPV was the probability that an adrenal mass with a negative test is truly a nonadenoma. *P* value <0.05 was considered statistically significant.

## RESULTS

### Patient Characteristics

Patient age, gender, and perioperative comorbidities were comparable between the adenomas and nonadenomas (pheochromocytoma or malignancy). There was a significant difference in the presence of classic pheochromocytoma symptoms between pheochromocytomas and other masses (adenoma and malignancy) (57.9% versus 13.8%, *P* < 0.001 and 57.9% versus 10.0%, *P* = 0.002, respectively). About 40% of patients with pheochromocytomas had no classic hyperadrenergic symptoms (Table 1).

**TABLE 1:**
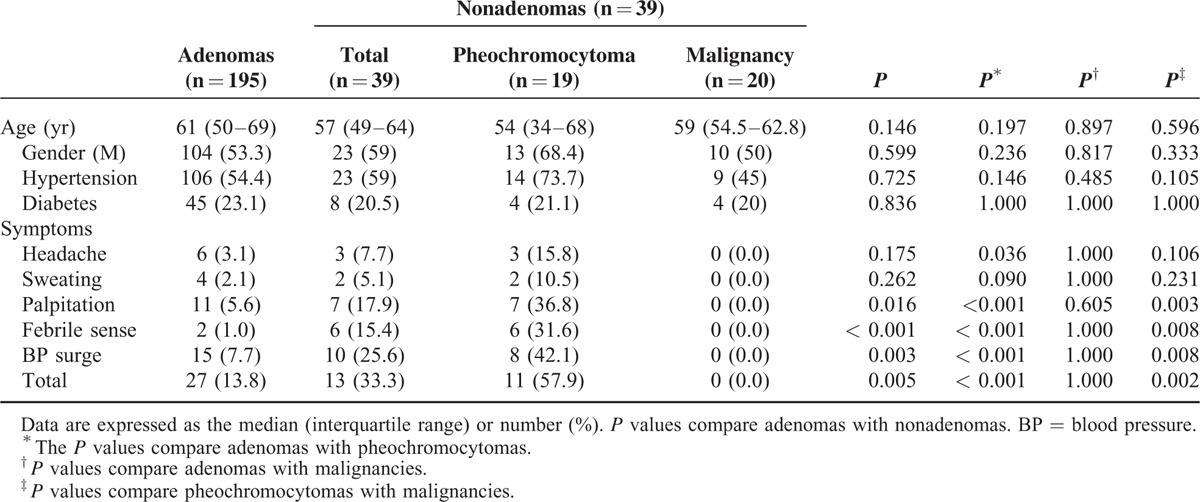
Characteristics of Patients With Adenomas and Nonadenomas

### Tumor Size

**FIGURE 2:**
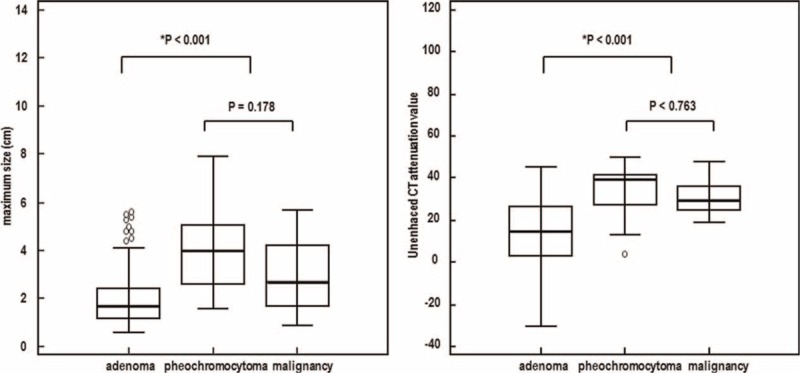
Maximum diameter of adrenal masses and attenuation values of unenhanced CT for different adrenal masses. The bold lines represent medians, the bottom of the box indicates the first quartile, the top of the box indicates the third quartile, and whiskers indicate the minimum and maximum non-distant values. Circles represent distant or extreme values. ∗*P* < 0.05 compared with other groups.

The maximum diameter of the adenomas was significantly smaller than that of nonadenomas (median [interquartile range]; 1.7 [1.2–2.4] cm versus 3.5 [2.2–4.8] cm, *P* < 0.001). There was no difference in the maximum diameter between pheochromocytomas and malignancies (4.0 [2.4–5.2] cm versus 2.7 [1.7–4.5] cm, *P* = 0.178) (Table 2). Differences in the maximum diameter among the individual histologic groups are shown in Figure 2.

**TABLE 2:**
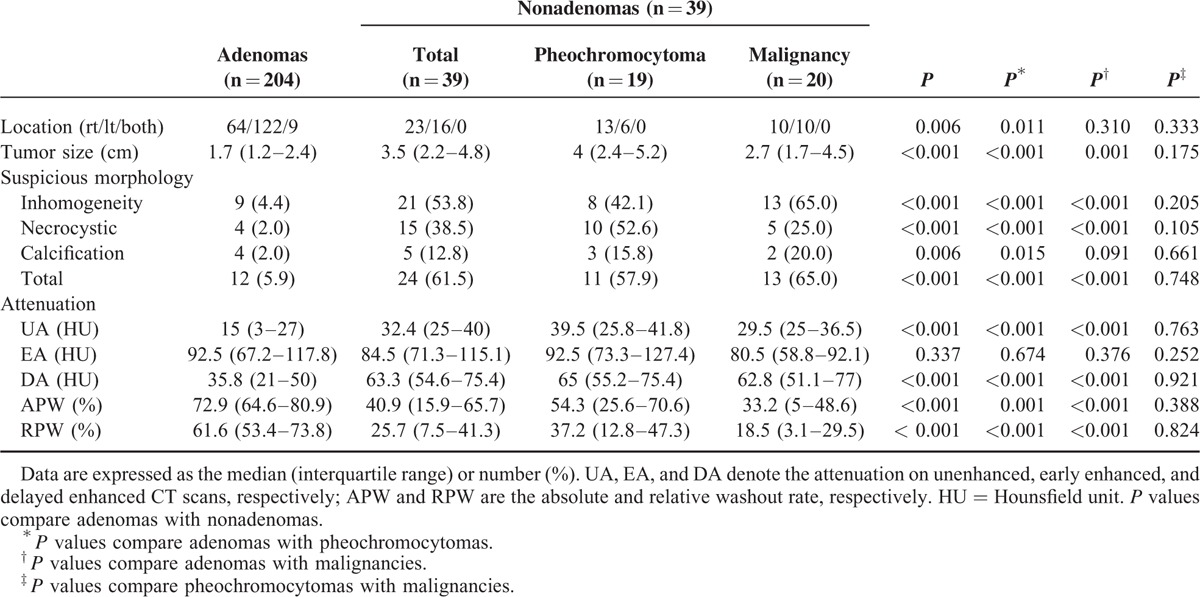
Radiologic Characteristics in Patients With Adrenal Adenomas and Nonadenomas

### Morphologic Criteria

The frequency of suspicious morphology (inhomogeneity, necrotic or cystic changes, and calcifications) was significantly less in adenomas than in nonadenomas (5.9% versus 61.5%, *P* < 0.001). There was no difference in the frequency of suspicious morphology between pheochromocytomas and malignancies (57.9% versus 65%, *P* = 0.748) (Table 2).

### Attenuation Value (Hu)

The attenuation values of unenhanced, early enhanced, and delayed enhanced CT are summarized in Table 2 and Figure 2. The attenuation values of unenhanced and delayed enhanced CT of adenomas were lower than those of nonadenomas (15 [3–27] HU versus 32.4 [25–40] HU, *P* < 0.001 and 35.8 [21–50] HU versus 63.3 [54.6–75.4] HU, *P* < 0.001, respectively), whereas the attenuation value of early enhanced CT was not different between adenomas and nonadenomas. There was no difference in the attenuation value of unenhanced and delayed enhanced CT between pheochromocytomas and malignancies (39.5 [25.8–41.8] HU versus 29.5 [25–36.5] HU, *P* = 0.763 and 65 [55.2–75.4] HU versus 62.8 [51.1–77] HU, *P* = 0.921, respectively) (Table 2, Fig. 2).

### Contrast Material Washout on Delayed Enhanced CT

The APW and RPW of adenomas were higher than those of nonadenomas (72.9 [64.6–80.9]% versus 40.9 [15.9–65.7]%, *P* < 0.001 and 61.6 [53.4–73.8]% versus 25.7 [7.5–41.3]%, *P* < 0.001, respectively). There were no differences in the APW and RPW between pheochromocytomas and malignancies (54.3 [25.6–70.6]% versus 33.2 [5–48.6]%, *P* = 0.388 and 37.2 [12.8–47.3]% versus 18.5 [3.1–29.5]%, *P* = 0.824, respectively) (Table 2).

### Sensitivity and Specificity

The sensitivity, specificity, PPV, and NPV of various CT phenotypes for distinguishing adenomas from nonadenomas are summarized in Table 3. The cutoff value of tumor size was set to 3 cm because adrenal masses larger than 3 cm are likely to be functional tumors.^14^ The cutoff values of UA, APW and RPW were decided by the previous diagnostic criteria of adenomas (UA ≤ 10 HU, APW ≥ 60%, or RPW ≥ 40%).^15^

**TABLE 3:**
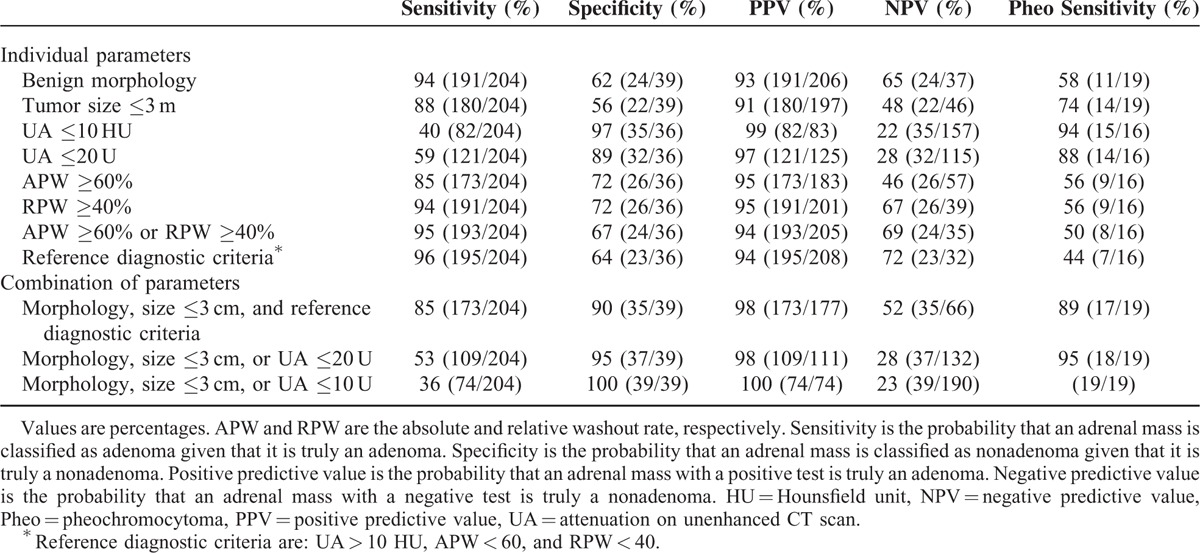
Radiologic Parameters Distinguishing Nonadenoma From Adenomas

A UA ≤10 HU was the single most specific phenotype for excluding pheochromocytomas and malignancies (specificity: 97%).

The purpose of our study was to exclude 100% of pheochromocytomas in the preoperative setting. Therefore, we tried various combinations of CT phenotypes to obtain higher specificity. A combination of tumor size ≤3 cm, UA ≤10 HU, and absence of suspicious morphology had 100% specificity for the exclusion of pheochromocytomas and malignancies (Table 3).

### Biochemical Tests and Intraoperative Hemodynamics

The 24-hour total urinary VMA testing was performed in 182/234 patients (77.8%). There were 5 false-positive and 6 false-negative results (sensitivity 68.4%, specificity 96.9%, PPV 72.2%, NPV 96.3%). Twenty-four hour urinary or plasma fractionated metanephrine tests were performed in 198 of 234 patients (84.6%). The urine or plasma fractionated metanephrine test showed 100% sensitivity (specificity 96.1%, PPV 73.1%, NPV 100%) (Table 4). However, 2 pheochromocytoma patients had an equivocal elevation in fractioned metanephrines. They had classic pheochromocytoma symptoms and could be diagnosed by imaging phenotypes obtained from adrenal-protocol CT (suspicious morphology, high attenuation on the unenhanced image, and low washout value) in contrast to the uncertain results of the biochemical study.

**TABLE 4:**
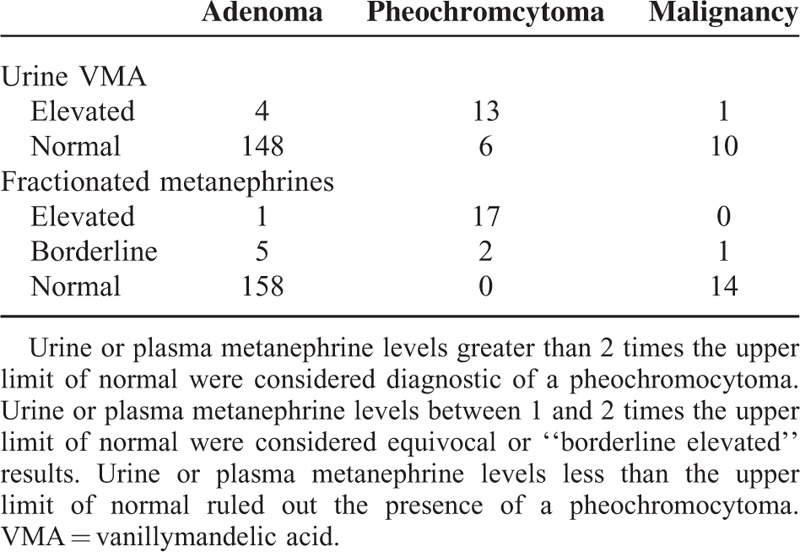
Biochemical Tests for Pheochromocytomas

Eighteen of 19 pheochromocytoma patients received antihypertensive medications preoperatively (doxazosin or phenoxybenzamine 15/19, beta blocker 5/19, calcium channel blocker 3/19, diuretics 1/19, no medication 1/19). The intraoperative hemodynamics significantly differed between patients with pheochromocytomas and nonpheochromocytomas. The administration of vasoactive drugs was more common in pheochromocytomas and maximum intraoperative SBP and heart rate were significantly higher in pheochromocytomas. However, 80% of pheochromocytoma patients did not have hypertensive crisis (systolic blood pressure [SBP] ≥200 mm Hg) during the operation (Table 5). There were no cardiovascular or cerebral complications perioperatively in pheochromcytoma patients.

**TABLE 5:**
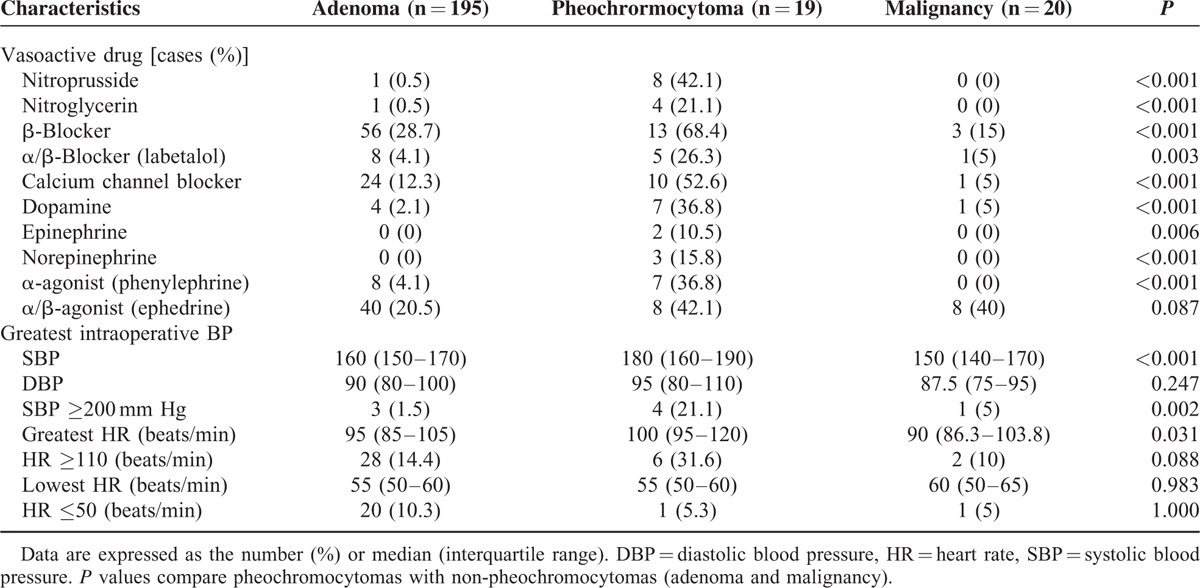
Intraoperative Characteristics: Intraoperative Use of Vasoactive Drugs and Hemodynamics

## DISCUSSION

An aging population and the widespread use of CT and MRI have resulted in the more frequent detection of incidental adrenal masses (AI). When an AI is found, it is essential to exclude pheochromocytoma in the preoperative setting. In this study, we found that a combination of an unenhanced attenuation value on CT (UA) ≤10 HU, size ≤3 cm, and absence of suspicious morphology (inhomogeneity, calcification, necrocystic change) could exclude pheochromocytomas with 100%. These features can be determined using the CT scan that patients already have and do not require contrast media or washout study.

Tumor shape and homogeneity can differentiate adrenal adenomas from nonadenomas. Benign adenomas are usually oval, homogeneous, and well-demarcated lesions.^16^ Pheochromocytomas usually appear round and smooth, but may include cystic, necrotic, or even hemorrhagic areas. In our cases, pheochromocytomas and malignancies showed more suspicious morphological features (inhomogeneity, calcification, necrocystic change) than adenomas. Although these features are helpful in the characterization of a mass, they could not rule out pheochromocytomas and malignancies (specificity: 62%).

Traditionally, size has been considered a very important parameter for AIs.^17^ The transformation rate of nonfunctional adrenal masses to functional tumors seems to be higher in adrenal masses greater than 3 cm in size.^14^ Our results showed that pheochromocytomas and malignancies are significantly larger than adenomas, in accordance with previous studies.^18,19^ However, when using size ≤3 cm as the criteria of adenoma, 26% of pheochromocytomas and 40% of malignancies escaped detection (specificity: 56%).

Another CT feature used to distinguish adenomas from nonadenomas is intracellular lipid content. The abundant intracytoplasmic fat in adenomas results in a lower attenuation value on unenhanced CT (UA) than nonadenomas.^19,20^ A UA ≤10 HU is suggestive of an adrenal adenoma.^19,21^ Pheochromocytomas are characterized by a high UA (>20 HU) and only very rarely have high lipid content.^22,23^ Sane et al^24^ reported that all 9 pheochromocytomas were >10 HU on unenhanced CT in their 146 AI cases and suggested 10  HU as a 100% exclusion threshold for pheochromocytoma. However, previous studies report pheochrmocytomas with UA less than 10 HU.^3,23^ In our study, most pheochromocytomas were characterized by a UA greater than 10 HU, but 1 symptomatic pheochromcytoma in our study showed 4 HU on unenhanced CT (specificity: 97%).

Nonadenomas exhibit disrupted capillary permeability, with a prolonged retention of contrast material in the effective extracellular space. Therefore, attenuation on delayed enhanced CT (DA) is highly accurate for differentiating nonadenomas from adenomas.^11,21,25^ Because DA varies according to the institutions’ contrast media protocol, contrast washout data (APW and RPW) are more preferred.^21,25,26^ Most investigators use a guideline of APW ≥60% or RPW ≥40% on a 15-minute delayed scan.^21^ This guideline showed low specificity for the exclusion of pheochromocytomas and malignancies in our study (specificity: 67%). This discrepancy comes from that most previous studies used washout data to detect malignancy not pheochromocytoma.^21,25,27–29^

Our priority was to exclude pheochromocytomas 100% as a preoperative screening test. A UA ≤10 HU was the single most specific phenotype. When size ≤3 cm and absence of suspicious morphology were added to UA, the specificity to exclude pheochromocytomas increased to 100%. The previously used reference diagnostic criteria (UA ≤10 HU, APW ≥60%, or RPW ≥40%)^15^ showed low specificity (64%) and high sensitivity (96%). As we mentioned before, these criteria were used to detect malignancies, therefore do not seem to be adequate for the preoperative pheochromocytoma screening.

The classic symptoms of pheochromocytoma are well known, but the absence of these features cannot exclude the presence of a pheochromocytoma.^8,30^ In our study, 30% of patients with pheochromocytoma were normotensive before surgery and 40% had no symptoms.

Traditional biochemical tests for pheochromocytoma include measurements of urinary VMA, and urinary or plasma fractionated metanephrines.^9,31^ VMA is not routinely used as an initial test anymore because of poor sensitivity.^9^ Consistent with this finding, the sensitivity of VMA in the current study was low (70%) and inadequate for pheochromocytoma exclusion. The sensitivity of metanephrine was previously reported to be 96% to 99%.^9^ In the current study, the sensitivity was 100%.

However, biochemical work-ups have several shortcomings. In a study by Yu et al,^8^ approximately 50% of patients with small pheochromocytomas exhibited modestly abnormal or even normal test results, even though most of these small tumors exhibited positive imaging characteristics. In our study, 2 patients with pheochromocytoma with a borderline elevation in urine or plasma fractionated metanephrine had classic pheochromocytoma symptoms and could be diagnosed by imaging phenotypes. Furthermore, obtaining an optimal specimen for biochemical study is not easy. Urinary collection for 24 hours is potentially incomplete and inconvenient for patients.^9^ Blood samples can be taken at any time, but false-positive cases are common. Exercise, posture, food, stress, and medications can result in modestly elevated metanephrine, bringing confusion to diagnosis.^9^ False-positive findings can be corrected by benign characteristics on CT findings.

For limitations, we followed our patients for 6 months, and it is possible that the diagnosis might have changed after this period. The diagnosis of adrenal adenoma was not histologically or biochemically verified in all cases because imaging-based diagnosis of adenoma is now the standard of care and it is considered unnecessary and unethical to perform biopsies to confirm adenoma.^21,25,27^ The combination of 3 imaging phenotypes (size ≤3 cm, UA ≤10 HU, and absence of suspicious morphology) could exclude pheochromocytomas with 100%. However, these criteria could not differentiate pheochromocytomas from adrenal malignancy because they share similar imaging phenotypes on CT, which is also reported in a previous study.^22^ Other imaging modalities and biochemical tests are required to differentiate pheocrhomocytoma from malignancy.

In conclusion, CT can be used as a screening tool for pheochromocytoma by combining 3 imaging phenotypes: size ≤3 cm, attenuation value on unenhanced CT ≤10 HU, and absence of suspicious morphology, and may substitute for biochemical testing in the preoperative setting. However, it remains still necessary to take a careful history and perform a physical examination, focusing on the signs and symptoms suggestive of adrenal hyperfunction. Also, confirmation of CT-based diagnosis by a following biochemical test would still be necessary to give the patient and clinician comprehensive information and confidence on their AI.
